# Dental Prophylaxis of Employes, Metropolitan Ins. Co.

**Published:** 1922-06

**Authors:** Thaddeus P. Hyatt


### DENTAL PROPHYLAXIS OF EMPLOYES, METROPOLITAN INS. CO.

THADDEUS P. HYATT, D.D.S.

The relation of dental defects to systemic conditions has been very thoroughly investigated and written about during the past few years. As is usual with the advent of new ideas, two schools of thought were quickly developed.

One school advocated extremely radical procedures, such as the ruthless extraction of all dental organs open to the slightest suspicion; the other school, ultra conservative, refused to accept the evidence shown by the remarkable restoration to health of many persons, after dental infection had been removed.

We must not allow the battle smoke of this hotly contested controversy regarding dental infection to blind us to the more important consideration of the part played in health or illness by unclean and unsanitary mouths.

It should be obvious to all that if pure water and clean wholesome food are really important factors for health, it is highly desirable to maintain the purity of the water and the wholesomeness of the food until it reaches the digestive apparatus of the individual, where it will be changed into a form that will be assimilated easily and thus sustain life and health.

It is here that the importance of mouth hygiene comes in. Of what avail is it to put pure wholesome food into a mouth filled with the decomposing debris of previous meals—of a mouth with diseased or broken down teeth?

Should it be surprising, then, that a procedure which brings about a continued clean and hygienic condition of the mouth should result in an improved physical well-being?

Careful and scientific investigation by Professor Gies, and Professor Kligler of Columbia University, shows that there is a reduction in number of at least 495 million bacteria per mm. in a fairly clean mouth as compared with a dirty one. This did not take into consideration a clean mouth.

It is safe to say the bacteria found in a clean mouth are practically harmless, while the bacteria that grow and multiply in a dirty mouth become virulent and dangerous. The long continued presence of bacterial masses in and around the teeth bring about inflammation of the gum tissues. Normal, healthy gums are practically impervious to bacterial invasion. But inflamed gums become open gateways for the entrance of harmful bacterial life into the blood-stream of the body. Professor Jordan in "General Bacteriology" says:

"The portal by which bacteria gain entrance to the human body is of great importance in determining whether or not disease shall occur. Typhoid bacilli rubbed into the abraded skin may give rise to no reaction of importance, while the same micro-organisms, if swallowed, may cause fatal infection. Conversely, virulent streptococci, when swallowed, may cause no harmful effffects, while the same bacteria rubbed into the skin may give rise to a severe reaction."

The question of gum condition is therefore of great importance, as the human mouth always contains a large number of streptococci, and streptoccus infection is made possible when the soft tissues of the mouth become badly inflamed.

Periodical prophylactic dental treatment must necessarily include X-ray examination, for it is only by the aid of the X-ray that we are enabled to ascertain the presence of abnormal conditions surrounding the roots of teeth.

In the Metropolitan Life Insurance Company's dental clinic, two cleansings of the mouth are given each year and X-ray examinations made of all teeth having crowns, bridges, or teeth with non-vital pulps. The work has been continued for a sufficient time to show certain results. Those who have come regularly twice a year since 1915 have the highest per cent of clean mouths and the lowest per cent of unclean mouths. Furthermore, the percentage of clean mouths has increased year by year and the percentage of unclean mouths has decreased year by year. This is true of all classes.

The classes have been designated as follows: Those who started in 1915 and have continued to come twice each year are called "Class 1." Those who started in 1916, "Class 2," etc.

Professor Gies' research the last three years shows considerable reduction in the number of bacteria in fairly clean mouths as compared with unclean mouths, we shall readily see that there probably has been a very great reduction in the number of bacteria in the mouth of the employes examined at the Metropolitan Life Insurance Company's clinic. This fact may be accountable for the following rule issued by the president of the Company in 1919, making dental examination a requirement:

The services rendered by the dental division since its establishment in 1915 have been so curative of impaired health conditions, and so permanently helpful to the employes who have taken advantage of the opportunities offered, that henceforth every home office employe will be required to undergo examination and cleansing of the teeth in the home office dental division twice a year. If the service of the family dentist is desired rather than that of the home office dentist, such examination and cleansings by him will be accepted, but they must be procured without expense to the Company and the employe must furnish a satisfactory certificate from him that the required work has been done." (Signed) HALEY FISKE, *President,* Metropolitan Insurance Co.

